# CD8 cytotoxic T-cell infiltrates and cellular damage in the hypothalamus in human obesity

**DOI:** 10.1186/s40478-023-01659-x

**Published:** 2023-10-09

**Authors:** Jared T. Ahrendsen, Yi Nong, Yuda Huo, Jasmine Steele, Matthew P. Anderson

**Affiliations:** 1 Department of Pathology, Beth Israel Deaconess Medical Center and Harvard Medical School, Boston, Massachusetts USA; 2 Department of Neurology, Beth Israel Deaconess Medical Center and Harvard Medical School, Boston, Massachusetts USA; 3 Present Address: Department of Pathology, Northwestern University Feinberg School of Medicine, Chicago, IL USA; 4 Present Address: Neuroscience Therapeutic Focus Area, Regeneron Pharmaceuticals, Tarrytown, NY USA

**Keywords:** Obesity, Hypothalamus, CD8 T cell, Inflammation

## Abstract

Rare cases of paraneoplastic obesity in children suggest sporadic obesity might also arise from an adaptive immune cell-mediated mechanism. Since the hypothalamus is a central regulator of feeding behavior and energy expenditure, we quantified lymphocytic inflammation in this region in a cohort of obese and non-obese human post-mortem brains. We report that CD8-positive cytotoxic T-cells are increased in hypothalamic median eminence/arcuate nucleus (ME/Arc) and bed nucleus of the stria terminalis in 40% of obese compared to non-obese patients, but not in other hypothalamic nuclei or brain regions. CD8 T-cells were most abundant in individuals with concurrent obesity and diabetes. Markers of cytotoxic T-cell induced damage, activated caspase 3 and poly-ADP ribose, were also elevated in the ME/Arc of obese patients. To provoke CD8 cytotoxic T-cell infiltrates in ventromedial region of hypothalamus in mice we performed stereotactic injections of an adeno-associated virus expressing immunogenic green fluorescent protein or saline. AAV but not saline injections triggered hypothalamic CD8 T-cell infiltrates associated with a rapid weight gain in mice recapitulating the findings in human obesity. This is the first description of the neuropathology of human obesity and when combined with its reconstitution in a mouse model suggests adaptive immunity may drive as much as 40% of the human condition.

**Supplementary Information:**

The online version contains supplementary material available at 10.1186/s40478-023-01659-x.

## Introduction

Obesity, a result of dysregulated energy balance, causes significant morbidity in affected individuals and substantial healthcare-related expenditures worldwide [1]. The pathogenesis of human morbid obesity is not fully understood with evidence suggesting influences from environmental, genetic, individual behavior, and co-morbid conditions [2, 3].

The hypothalamus is a key central regulator of several homeostatic functions, including feeding behavior and energy expenditure [3]. Accumulating evidence implicates hypothalamic dysfunction in generating an obese phenotype [4]. Reactive astrogliosis and microgliosis has been observed in the hypothalamus in response to high-fat diet in rodent models [5–7]. Viral infection of hypothalamic neurons can induce an obese phenotype in mice [8, 9].

Brain imaging studies have demonstrated an association between obesity and various correlates of hypothalamic gliosis and dysfunction [10–12], particularly in the ventral medial hypothalamus [5, 13]. Limited post-mortem studies have also suggested hypothalamic microgliosis and astrogliosis as potential contributors to human obesity [14, 15]. Destructive lesions of the hypothalamus are known to cause obesity, such as tumors of the sellar region, radiation injury, and hypothalamic involvement by sarcoidosis [16]. Similarly, in children with Rapid-onset Obesity with Hypothalamic dysfunction, Hypoventilation, and Autonomic Dysreguation (ROHHAD) syndrome, there is evidence for paraneoplastic “autoimmune” inflammation causing hypothalamic injury and obesity [17–19].

However, a human tissue-based study has not been performed to systematically evaluate whether an adaptive cellular immune response in the hypothalamus can be found in association with obesity. Therefore, we quantified lymphocyte-related inflammation in a large cohort of human post-mortem brains from obese and non-obese decedents. We also utilize a mouse model in which an immunogenic adeno-associated virus expressing green fluorescent protein peptides under a general cytomegalovirus promoter (AAV-CMV-GFP) is injected into the ventromedial hypothalamus to recapitulate the pathology observed in human brains.

## Materials and methods

### Human samples

Post-mortem human brain tissues were collected from autopsy cases performed at Beth Israel Deaconess Medical Center from the years 2018–2021 in which specific consent for research was obtained from the decedent’s next of kin and/or health care proxy. Clinical parameters were tabulated from the patient’s medical chart, including age, sex, height, weight, and co-morbid medical conditions. Body mass index was calculated from recorded height and weight data obtained during autopsy. Exclusion criteria included: global cerebral ischemia, localized hypothalamic infarction, or tumor involvement of the hypothalamus. In total, tissue was collected from 50 subjects. Standard tissue sections were obtained from all cases, including the prefrontal cortex, hippocampus with adjacent temporal cortex at the level of the lateral geniculate nucleus, calcarine cortex, basal ganglia at the level of the anterior commissure, thalamus at the level of the subthalamic nucleus, midbrain to include substantia nigra, pons to include locus coeruleus, medulla to include the inferior olives, and cerebellum to include dentate nucleus. The hypothalamus for each case was entirely blocked and sectioned into approximately 3–5 mm tissue slabs. Formalin-fixed paraffin embedded tissue blocks were generated and processed according to standard institutional procedures. Tissue sections were stained with hematoxylin and eosin with luxol fast blue (H&E/LFB) for histologic examination. All histologic sections were examined by two board certified neuropathologists (JTA, MPA).

Immunohistochemistry was performed according to standard institutional procedures with the following antibodies: CD3, CD4, CD8, CD20 (Dako, Agilent Pathology Solutions), activated caspase 3 (clone C92-605, BD Biosciences), and poly(ADP-ribose) [PAR, clone 10 H, Enzo Life Sciences]. Multiple photomicrographs of each brain region of interest were taken. Quantification was performed manually utilizing ImageJ software. All image acquisition and quantification were performed blinded to BMI status.

### Mouse experiments

The Harvard Medical Area Standing Committee on Animals and the Institutional Animal Care and Use Committee of Beth Israel Deaconess Medical Center approved all mouse protocols. Mice were housed at the Center for Life Sciences barrier animal facility in sex-matched groups of 3–5 with ad libitum food and water access.

Adult (approximately 13 weeks old) female FVB mice were used for all experiments. Anesthesia was induced in a chamber with isofluorane/oxygen. The mice were then placed into a stereotaxic frame fitted with a continuous isofluorane delivery system. A single midline vertical scalp incision revealed skull landmarks. Stereotactic measurements were used to make 0.7 mm wide burr holes over the entry point for bilateral viral injections directed at the arcuate nucleus (0 deg, AP -1.7 mm, ML +/- 0.25 mm, DV -5.85 mm), all relative to bregma. 1 µL of virus (*AAV9-CMV-GFP*) was infused at 0.2 µL/min through a 33-gauge Hamilton needle connected to an automated infusion pump. Following each infusion, the needle remained in place for 5 min. The incision was sutured closed and a single injection of 10 mg/kg meloxicam dissolved in saline was administered intraperitoneally for perioperative analgesia. Accurate targeting to arcuate was confirmed by immunohistochemistry. Mouse body weights were measured one day before the virus injection, the day of the virus injection, and every two days after the virus injection.

Mice were sacrificed for histologic analysis 6 weeks after the virus injection in CO_2_ chamber followed by intracardiac perfusion sequentially with 20 mL PBS first and 20 mL 4% paraformaldehyde (PFA) in PBS. Brains were removed and further fixed in the 4% PFA in PBS overnight at 4 °C. Fixed brains were thoroughly rinsed with PBS and kept in PBS with 0.1% sodium azide at 4 °C, embedded in 2% agarose gel in PBS, and mounted onto the Compresstome (Precisionary Instruments). 100 μm thick coronal brain slices including the arcuate nucleus (Arc) were selected for further immunohistochemistry. Slices were permeabilized with 1% Triton X-100 in PBS (PBST) overnight at 4 °C, followed by blocking in 20% bovine serum albumin with PBST for 1 h at room temperature. Primary rat anti-mouse CD3 (ThermoFisher, 1:200) and rat anti-mouse CD8 (ThermoFisher, 1:200) antibodies were prepared in 1% goat serum with PBST, applied to brain slices and incubated overnight at 4 °C. Secondary goat anti-rat antibody conjugated with Alexa Fluor 568 (ThermoFisher, 1:400) was then prepared in PBS and incubated with primary antibody labelled brain slices for 2 h at room temperature. Nuclei were labeled with 1 µg/mL Hochest 33,342 (ThermoFisher) in PBS for 5 min. Images were acquired with Leica DM6 confocal microscopy (Leica Microsystems, Germany) with 10x objective by Leica Application Suite X software. Exposure time was fixed throughout the imaging process. CD3- and CD8-positive cells were quantified by particle analysis function with the same selection size to avoid bias.

### Statistics

Statistical analyses were performed using GraphPad Prism software version 9.5.0. Data are presented as the mean ± standard error of the mean. Two-sided student’s t-test or two-way ANOVA was used to compare the means between groups, with a p-value of less than 0.05 considered statistically significant. Correction for multiple comparisons were not performed. The data that support the findings of this study are available from the corresponding author, upon reasonable request.

## Results

Post-mortem brain tissue was examined from 50 patients who died between the years 2018–2021. Height and weight data were collected at autopsy, from which body mass index (BMI) was calculated. Obesity was defined as BMI ≥ 30.0 kg/m^2^, with 25 patients having a non-obese phenotype (BMI < 30.0 kg/m^2^, control) and 25 patients having an obese phenotype (BMI ≥ 30.0 kg/m^2^, obese). Amongst the 25 non-obese patients, there were 14 males and 11 females, with a mean age of 68.2 years at death (range 25–97 years). Amongst the obese patients, there were 16 males and 9 females, with a mean age of 68.7 years at death (range 40–83 years). A clinical history of diabetes was documented for 5 non-obese patients and 18 obese patients. The cause of death was tabulated for each patient, including cardiac, pulmonary, cancer-related, infections, cerebrovascular accident, and liver/gastrointestinal, which did not differ between control and obese patients (Table 1).

**Table 1:** Clinical characteristics human post-mortem cohort

	Non-obese	Obese
Count (n)	25	25
M:F	14:11	16:9
Mean age at death [range], years	68.2 [25–97]	68.7 [40–83]
Body mass index (BMI)		
<18.5, underweight	1	0
18.5–24.9, normal weight	11	0
25.0-29.9, overweight	13	0
30.0-39.9, obese	0	13
≥ 40.0, morbidly obese	0	12
Clinical history of diabetes (%)	5 (20%)	12 (48%)
Cause of death		
Cardiac	7	6
Pulmonary	1	1
Cancer	3	4
Infection	7	7
Cerebrovascular	3	3
Liver/gastrointestinal	4	4

**Fig. 1:**
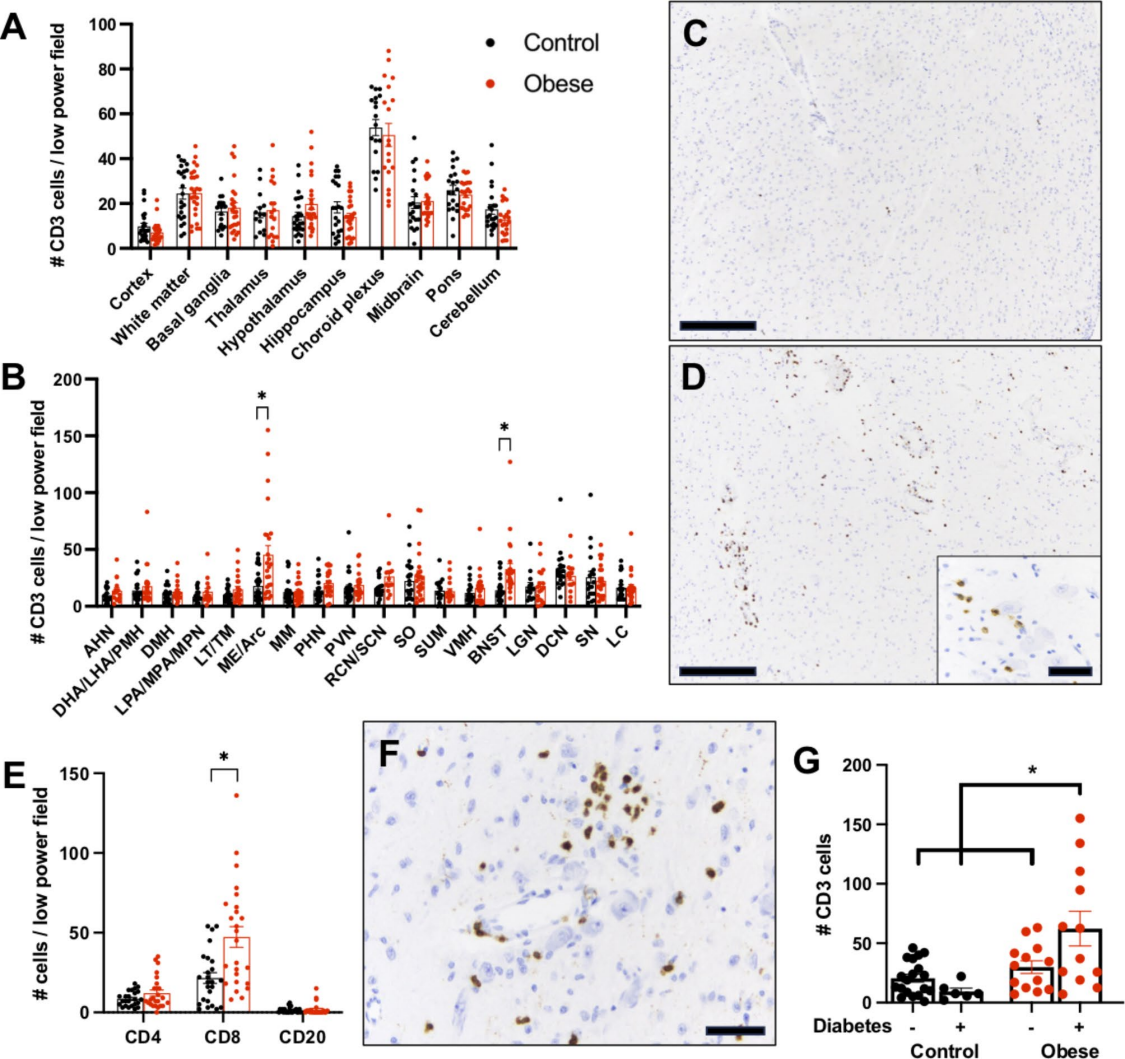
T cell infiltrates are increased in hypothalamic arcuate/medial eminence and in bed nucleus of the stria terminalis of obese humans. CD3-positive cells were quantified in standardized post-mortem brain tissue sections from control and obese humans **(A)**. CD3-positive T-cells in various hypothalamic regions and other non-hypothalamic brain nuclei from control and obese humans **(B)**. Representative CD3 immunohistochemistry in the Arc/ME of a non-obese **(C)** and obese **(D)** individual. Note the close apposition of CD3-positive T-cells to hypothalamic neurons (D, inset). Quantification of CD4, CD8, and CD20 inflammatory cells in the Arc/ME from control and obese individuals **(E)**. Representative CD8 immunohistochemistry from the Arc/ME from an obese human **(F)**. CD3-positive cells in the Arc/ME of non-obese/obese individuals with and without a clinical history of diabetes **(G)**. Scale bars: A,D = 500 μm, D(inset), F = 50 μm. * p-value < 0.05

To evaluate for the presence of cellular immunity in obesity, standard post-mortem brain samples were collected, including the entirety of the hypothalamus, and submitted for histologic examination following formalin fixation and paraffin embedding. CD3 immunostaining was performed on every examined brain region from obese and non-obese patients and the number of CD3-positive lymphocytes per low power field (4x objective) was counted (Fig. 1A, B). There was no difference in the number of CD3-positive lymphocytes between non-obese and obese patients in any of the following brain regions: cerebral cortex, subcortical white matter, basal ganglia, thalamus, hippocampus, hypothalamus (overall), choroid plexus, midbrain, pons, and cerebellum (Fig. 1A). As expected, the number of lymphocytes was greatest in the choroid plexus, and relatively low in other brain regions.

The hypothalamus is an anatomically complex brain region with multiple nuclei that perform variable and, in some instances, opposing roles, particularly in regard to feeding behavior and satiety. Therefore, we performed a detailed neuroanatomical analysis by counting CD3-positive lymphocytes in specific hypothalamic and non-hypothalamic nuclei. In the majority of examined nuclei, there was no difference in the number of CD3-positive lymphocytes between control and obese patients (Fig. 1B). However, there were significantly more CD3-positive lymphocytes in the arcuate/median eminence (Arc/ME) and bed nucleus of the stria terminalis (BNST) in obese (Fig. 1D) compared to non-obese cases (Fig. 1C). CD3-positive lymphocytes were observed around blood vessels, infiltrating into the brain parenchyma (Fig. 1D), and many CD3-positive lymphocytes were in close proximity to neuron cell bodies in the Arc/ME (Fig. 1D, inset).

To further examine the type of lymphocytes responsible for the increased inflammatory cell infiltrates in the Arc/ME of obese patients, we performed CD4, CD8, and CD20 immunostaining to quantify helper T-cells, cytotoxic T-cells, and B-cells, respectively. There was no difference in the number of CD4- or CD20-positive cells in the Arc/ME between obese and non-obese individuals. However, there were more CD8-positive cytotoxic T-cells in the Arc/ME of obese compared to non-obese individuals (Fig. 1E-F). Using the control patient with the highest level of CD8-positive T-cells in the Arc/ME as a threshold, there were 10 of 25 obese patients (40%) with increased CD8-positive T-cells in the ME/Arc.

Interestingly, obese individuals with a clinical history of diabetes demonstrated a greater increase of CD3-positive T-cells in the Arc/ME than obese individuals without diabetes or non-obese individuals regardless of diabetes history (Fig. 1G). Because gender-related dimorphism exists in many brain regions, including the BNST, we stratified CD3 counts by gender and obesity status. There was no statistically significant difference between male and female gender in the number of CD3-positive T-cells in either the Arc/ME or BNST with this collection of 25 obese and 25 control cases (Supplemental Fig. 1).

**Fig. 2:**
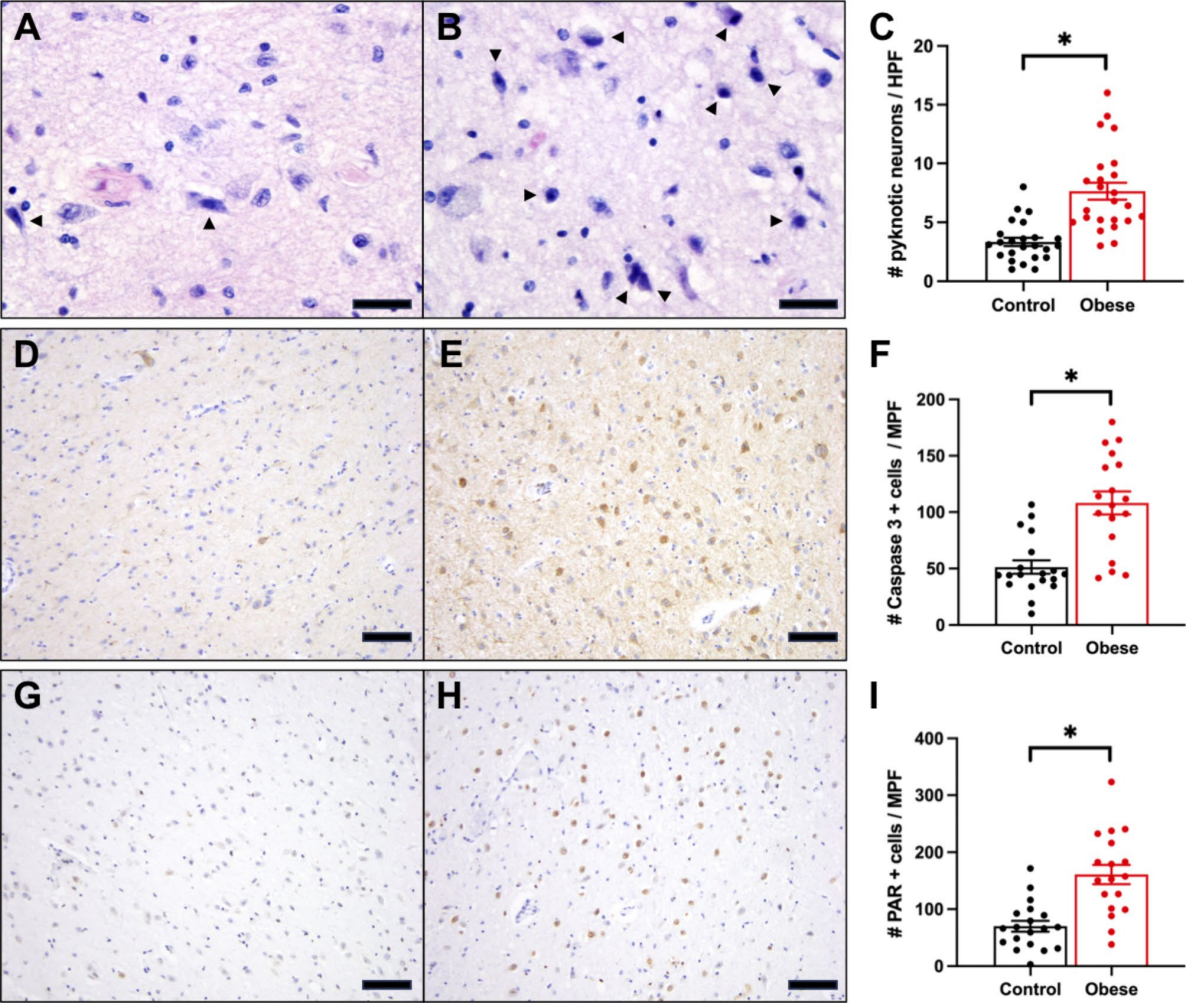
Neuronal response in CD8 T-cell infiltrated arcuate/medial eminence of obese humans. Histologic examination reveals increased numbers of neurons with shrunken cytoplasm with pyknotic nuclei (arrowheads) in the Arc/ME of obese **(B)** compared to non-obese **(A)** human brains. Representative immunohistochemistry for activated caspase 3 **(D, E)** and poly-ADP ribose (PAR; G, H) in the Arc/ME of non-obese **(D, G)** and obese **(E, H)** humans. Increased levels of neuron “injury” were observed in obese compared to non-obese groups **(C, F, I)**. Scale bars: A,B = 250 μm, D,E,F,G = 400 μm. * p-value < 0.05

We also examined the hypothalamus from each patient by routine H&E/luxol fast blue staining to look for possible histologic evidence of cell damage caused by the increased CD8 cytotoxic T-cells. Cytotoxic T-cells are known to induce neuronal damage in a variety of conditions, most notably in cortex of Rasmussen encephalitis T-cell infiltrates were associated with neurons displaying a condensed cytoplasm with loss of Nissl substance and hyperchromatic nuclei with obscured nucleoli [20]. We imaged multiple fields from the Arc/ME region in each case and control and counted the number of neurons with the histologic changes reported in this prior CD8 T-cell neuronal disease while blinded to BMI status. There were more of the “pyknotic” neurons per high power field in the Arc/ME of obese compared to non-obese individuals (Fig. 2A-C). We then performed immunohistochemistry with activated caspase 3 and poly-ADP ribose (PAR) antibodies, markers of cytotoxic T-cell mediated neuronal damage [21, 22]. As expected, we observed increased numbers of activated caspase 3-positive cells (Fig. 2D-F) and PAR-positive cells (Fig. 2G-I) in the Arc/ME of obese compared to non-obese individuals.

**Fig. 3:**
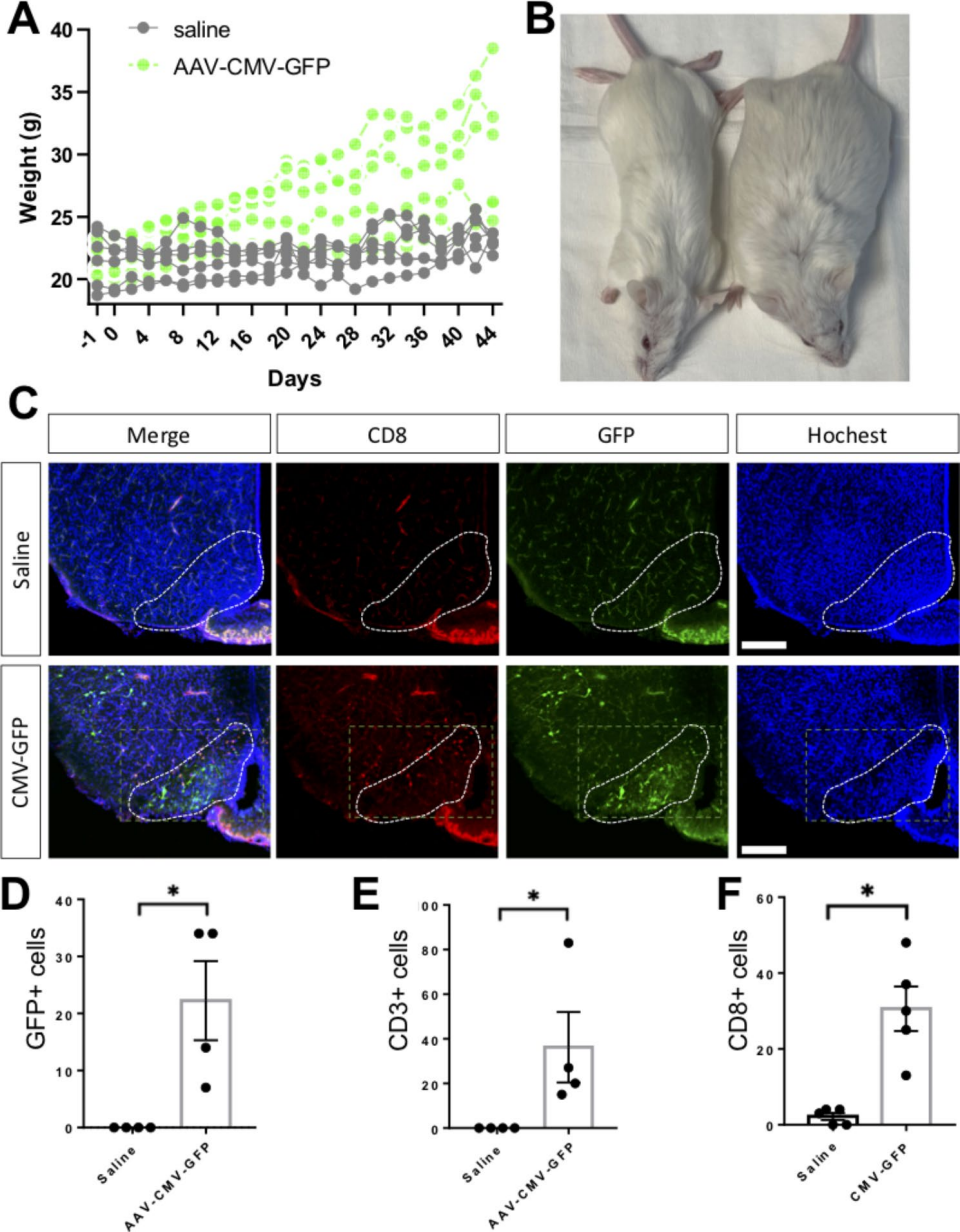
Stereotactic injection of AAV viral vector expressing Green Fluorescence Protein (GFP) vs. saline in hypothalamic arcuate nucleus causes obesity in mice. Gradual gain of weight starting approximately 7 days after injection in AAV-CMV-GFP compared to saline injected mice **(A, B)**. Immunohistochemistry demonstrates increased CD8 cytotoxic T-cells in areas with AAV-encoded GFP expression (**C**, all photomicrographs identical scale; scale bars = 1 mm). Quantification reveals increased GFP, CD3, and CD8 antibody reactivity in the median eminence of AAV-CMV-GFP mice compared to controls (**D-F**, respectively). * p-value < 0.05

The human data above suggest CD8-positive cytotoxic T-cell infiltrates and neuronal injury responses in ventromedial hypothalamus may be sufficient to induce obesity. To test this hypothesis, we performed stereotactic injections of AAV-CMV-GFP or saline into ventromedial hypothalamus of adult mice. Mice injected with AAV-CMV-GFP gained weight starting approximately one week after injection and continued to gain weight until they were sacrificed 6 weeks after injection, whereas the weight of mice injected with saline remained relatively constant (Fig. 3A). AAV-CMV-GFP injection generated an obese phenotype not seen in saline injected mice (Fig. 3B). Histologic examination 6 weeks after injection revealed an increased number of CD8 immunoreactive cells in areas of GFP expression in the ventromedial hypothalamus of AAV-CMV-GFP injected mice (Fig. 3C). Quantification revealed increased CD3 and CD8-positive T-cells in the arcuate nucleus and median eminence of AAV-CMV-GFP compared to saline injected mice (Fig. 3D-F). These results suggest cytotoxic CD8 T-cell inflammation in ventromedial hypothalamus may be sufficient to induce obesity in mice recapitulating the obesity-associated CD8 T-cell infiltrates found in humans.

## Discussion

Here, we performed a detailed neuroanatomic analysis of adaptive immune cells in post-mortem human brain tissue from obese and non-obese individuals. We report increased numbers of CD3 and CD8 positive T-cells in the hypothalamic Arc/ME and BNST of obese compared to non-obese individuals. We also demonstrate neuronal responses previously reported in the CD8 T-cell rich epilepsy disorder, Rasmussen encephalitis (associated with epileptiform discharge of the neurons in cortex where the T-cells infiltrate). Neuronal responses to the CD8-rich T-cell infiltrate are found in the Arc/ME of obese individuals as assessed by histologic H&E and immunohistochemical staining with CD8 cytotoxic damage-induced marker antibodies. We recapitulated obesity and increased CD3 and CD8 T-cell infiltrates in mice by injecting AAV-CMV-GFP into ventromedial hypothalamus. Interestingly, one patient in our cohort underwent gastric bypass surgery for obesity, resulting in temporary weight loss. However, this patient subsequently regained weight (BMI of 40.8 at time of death) and demonstrated high levels of T-cell inflammation in the Arc/ME of the hypothalamus. Together, our data support the hypothesis that hypothalamic CD8 cytotoxic T-cell inflammation may promote as much as 40% of human obesity.

There are no studies reporting T-cell-mediated inflammation in the hypothalamus in human obesity. Two prior studies using post-mortem human tissue suggest a link between gliosis (astrocytic and microglial) in hypothalamus and obesity. Baufeld et al. examined tissues from 12 obese and 9 non-obese individuals and showed increased microgliosis in the arcuate nucleus of obese patients [14]. Similarly, Kreutzer et al. showed evidence of reactive gliosis (GFAP and Iba1 staining) in the hypothalamus of a single overweight individual compared to a single non-obese individual [15]. These studies are limited due to their small sample size, restricted neuroanatomical examination, focus on intrinsic astrocytic and microglial reactions, the lack of correlates to neuronal dysfunction. Our data expand upon these prior findings demonstrating CD8-rich T-cell inflammation in hypothalamus is associated with histopathologic evidence of neuronal injury in ventromedial region of hypothalamus in approximately a third of obese individuals most prominently in those with comorbid diabetes.

One question is whether T-cell inflammation in ventromedial region of hypothalamic is caused by, or is a result of, obesity. Prior studies have established that introducing a foreign protein such as green fluorescent protein (GFP) *via* AAV viral vector into a mammalian species can elicit a T-cell immune reaction against the cells expressing the foreign protein eventually resulting in a loss of those cells [23]. Herein, we used this feature to reconstitute in mice the T-cell inflammatory infiltrate in Arc/ME found in human obesity. This intervention was sufficient to induce obesity in mice. While there is evidence of neuronal damage, we suspect obesity might result from the effects of inflammatory mediators on neuronal function in this region of the hypothalamus; possibly neuronal circuit hyperactivity as suggested by the CD8 T-cell promoted epilepsy in Rasmussen’s encephalitis. Alternatively, there might be mechanisms unique to the feeding neuronal circuits: SOCS-3 is a downstream effector of cytokine signaling and has been shown to have a role in leptin resistance in the brain [24].

These data raise additional questions concerning the potential role and mechanisms whereby T cell-mediated inflammation in ventromedial region of hypothalamus might generate symptoms comorbid with obesity such as diabetes and hypertension in humans. Transplantation of wild-type hypothalamic progenitor neurons into the ventromedial region of hypothalamus of leptin deficient mice not only partially rescues the obese phenotype, but also hyperglycemia [25], demonstrating a physiologic link between hypothalamic function, obesity, and blood glucose levels.

We also identify increased T-cell inflammatory infiltrates in BNST of a smaller subset of obese compared to non-obese individuals with a trend for increased frequency in males (Supplementary Fig. 1). The BNST has recently been shown to modulate feeding behavior in response to a variety of systemic inflammatory signals [26] and there are direct synaptic connections between BNST and hypothalamus that regulate feeding behavior [27].

In conclusion, we demonstrate CD8 cytotoxic T-cell inflammation specifically in Arc/ME hypothalamic nuclei (and in BNST) in ~ 40% of obese compared to non-obese individuals. CD8 cytotoxic T-cells in hypothalamic Arc/ME associate with markers of neuronal injury. Finally, recapitulating the CD8 cytotoxic T-cell based inflammation in ventromedial region of hypothalamus in using AAV viral vector expressing GFP in mice is sufficient to induce obesity. The results provide the first description of a neuropathology of obesity that could motivate sampling and immunohistochemical staining of these brain regions during postmortem examinations of human obesity in clinical neuropathology practice.

### Electronic supplementary material

Below is the link to the electronic supplementary material.

Supplemental Fig. 1. T cell infiltrates are not different between male and female patients in hypothalamic arcuate/medial eminence (A) or in bed nucleus of the stria terminalis (B) of humans.

## Abbreviations

AHN: Anterior hypothalamic nucleus
BNST: Bed nucleus of the stria terminalis
DCN: Deep cerebellar nucleus
DHA/LHA/PMH: Dorsal hypothalamic area/lateral hypothalamic area/posteromedial hypothalamic nucleus
DMH: Dorsomedial hypothalamic nucleus
H&E/LFB: Hematoxylin and eosin/luxol fast blue
LC: Locus coeruleus
LGN: Lateral geniculate nucleus
LPA/MPA/MPN: Lateral preoptic area/medial preoptic area/medial preoptic nucleus
LT/TM: Lateral tuberal nuclei/tuberomammillary nucleus
ME/Arc: Median eminence/arcuate nucleus of hypothalamus
MM: Medial mammillary nucleus
PAR: Poly-ADP ribose
PHN: Posterior hypothalamic nucleus
PVN: Paraventricular nucleus
RCN/SCN: Retrochiasmatic nucleus/suprachiasmatic nucleus
SN: Substantia nigra
SO: Supraoptic nucleus
SUM: Supramammillary nucleus
VMH: Ventromedial hypothalamic nucleus

## Declarations

### Availability of data and material

Data available through the corresponding author.
