# Supplementary figures and images for: CD8 cytotoxic T-cell infiltrates and cellular damage in the hypothalamus in human obesity
Source: Acta Neuropathol Commun. 2023 Oct 9;11:163. doi: 10.1186/s40478-023-01659-x (PMC10563257; doi:10.1186/s40478-023-01659-x)

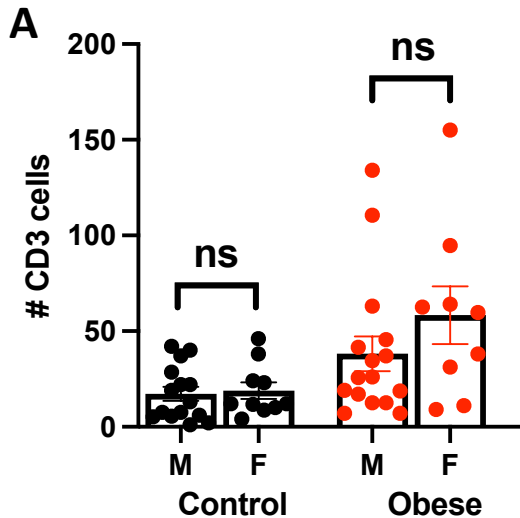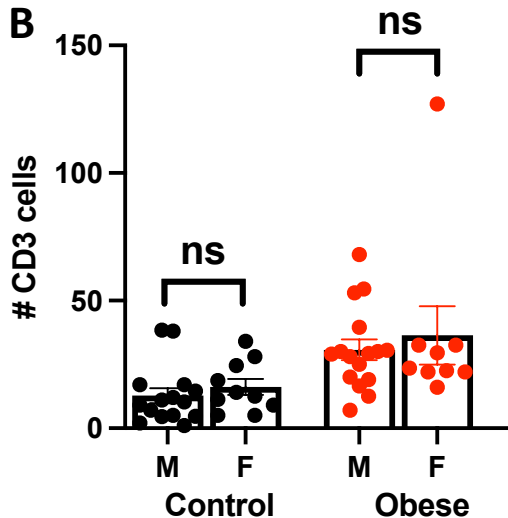

Supplement: Supplementary file 1 — Supplemental Fig. 1. T cell infiltrates are not different between male and female patients in hypothalamic arcuate/medial eminence (A) or in bed nucleus of the stria terminalis (B) of humans. [file 40478_2023_1659_MOESM1_ESM.pdf]
